# Cross-sectional Associations between Exposure to Persistent Organic Pollutants and Leukocyte Telomere Length among U.S. Adults in NHANES, 2001–2002

**DOI:** 10.1289/ehp.1510187

**Published:** 2015-10-09

**Authors:** Susanna D. Mitro, Linda S. Birnbaum, Belinda L. Needham, Ami R. Zota

**Affiliations:** 1 Department of Environmental and Occupational Health, Milken Institute School of Public Health, George Washington University, Washington DC, USA; 2 National Cancer Institute, National Institutes of Health, Department of Health and Human Services, Research Triangle Park, North Carolina, USA; 3 Department of Epidemiology, School of Public Health, University of Michigan Ann Arbor, Ann Arbor, Michigan, USA

## Abstract

**Background:**

Exposure to persistent organic pollutants (POPs) such as dioxins, furans, and polychlorinated biphenyls (PCBs) may influence leukocyte telomere length (LTL), a biomarker associated with chronic disease. In vitro research suggests dioxins may bind to the aryl hydrocarbon receptor (AhR) and induce telomerase activity, which elongates LTL. However, few epidemiologic studies have investigated associations between POPs and LTL.

**Objectives:**

We examined the association between 18 PCBs, 7 dioxins, and 9 furans and LTL among 1,330 U.S. adults from NHANES 2001–2002.

**Methods:**

We created three summed POP metrics based on toxic equivalency factor (TEF), a potency measure including affinity for the AhR: a) non–dioxin-like PCBs (composed of 10 non–dioxin-like PCBs; no AhR affinity and no TEF); b) non-ortho PCBs (composed of 2 non–ortho-substituted PCBs with high TEFs); and c) toxic equivalency (TEQ) (composed of 7 dioxins, 9 furans, 2 non–ortho-substituted PCBs, and 6 mono–ortho-substituted PCBs; weighted by TEF). We tested the association between each metric and LTL using linear regression, adjusting for demographics, blood cell count and distribution, and another metric with a different TEF (i.e., non-ortho PCBs and TEQ adjusted for non–dioxin-like PCBs; non–dioxin-like PCBs adjusted for non-ortho PCBs).

**Results:**

In adjusted models, each doubling of serum concentrations of non-ortho PCBs and TEQ was associated with 3.74% (95% CI: 2.10, 5.40) and 5.29% (95% CI: 1.66, 9.05) longer LTLs, respectively. Compared with the lowest quartile, the highest quartile of exposure was associated with 9.16% (95% CI: 2.96, 15.73) and 7.84% (95% CI: –0.53, 16.92) longer LTLs, respectively. Non–dioxin-like PCBs were not associated with LTL.

**Conclusions:**

POPs with high TEFs and AhR affinity were associated with longer LTL. Because many dioxin-associated cancers are also associated with longer LTL, these results may provide insight into the mechanisms underlying PCB- and dioxin-related carcinogenesis.

**Citation:**

Mitro SD, Birnbaum LS, Needham BL, Zota AR. 2016. Cross-sectional associations between exposure to persistent organic pollutants and leukocyte telomere length among U.S. adults in NHANES, 2001–2002. Environ Health Perspect 124:651–658; http://dx.doi.org/10.1289/ehp.1510187

## Introduction

Polychlorinated biphenyls (PCBs) and dioxins are ubiquitous persistent organic pollutants (POPs). PCBs were once widely manufactured and used as coolants or lubricants, whereas dioxins were unintentionally produced as industrial byproducts in a variety of commercial and industrial settings [Agency for Toxic Substances and Disease Registry (ATSDR) 1994, 1998]. Although PCB production and use were banned in the mid-1970s in the United States, most people continue to be exposed at low levels through food (ATSDR 1994, 1998, 2000). Exposure to dioxins and dioxin-like compounds is associated with overall cancer risk, although the carcinogenic mechanisms remain poorly understood [International Agency for Research on Cancer (IARC) 2012]. Dioxins may increase the risk for lung cancer, soft-tissue sarcoma, and non-Hodgkin lymphoma (IARC 2012). PCBs are also likely carcinogens and may raise cancer risk for melanoma as well as for cancers of the liver, intestines, and biliary tract (ATSDR 2000).

Telomeres are noncoding segments of DNA at the ends of chromosomes (Calado and Young 2009). Because telomeres are shortened with every cell division, eventually reaching a critical length that triggers cell senescence, they are considered a measure of cellular aging (Calado and Young 2009; Cong et al. 2002). Although telomeres typically shorten over the lifetime of a cell, under certain circumstances, telomeres may instead be lengthened. Telomerase, which is composed of subunits TERT (telomerase reverse transcriptase) and TERC (rRNA component of telomerase), can synthesize new telomere DNA sequences, thereby elongating telomeres (Cong et al. 2002). In normal somatic adult cells, telomerase expression is very low or undetectable, but telomerase is frequently expressed in immortalized or cancerous cells (Cong et al. 2002; Sarkar et al. 2006).

Leukocyte telomere length (LTL) is sometimes measured in epidemiological studies as a proxy for whole-organism telomere length. In epidemiological studies, short LTL has been associated with increased risk for a variety of chronic conditions, including cardiovascular disease (Haycock et al. 2014), Type II diabetes (Willeit et al. 2014), and metabolic syndrome (Révész et al. 2014). Therefore, it has been suggested that LTL may act as a measurable biomarker not only for cellular aging but also for organismal aging (Aubert and Lansdorp 2008).

Exposure to some environmental chemicals may affect LTL, and both positive and inverse associations have been reported in the literature. Exposure to cadmium, lead, polycyclic aromatic hydrocarbons, and traffic pollution have all been associated with short LTL (Hoxha et al. 2009; Zhang et al. 2013; Zota et al. 2015). Arsenic and particulate matter have also been associated with LTL, although the direction of the association depends on the dose or duration of exposure (Ferrario et al. 2009; Hou et al. 2012; Zhang et al. 2013). Finally, benzene exposure has been associated with increased LTL in occupationally exposed workers (Bassig et al. 2014). Because exposure to some toxic environmental chemicals is associated with increased LTL, the relationship between environmental chemicals, LTL, and overall health is likely complex.

Despite the evidence of associations with other environmental chemicals, only one epidemiologic study has investigated the relationship between PCBs and LTL. That study, a cross-sectional analysis of 84 Korean adults, reported an association between exposure to PCBs and longer LTL. However, the study did not account for the possible confounding effect of simultaneous exposure to multiple different PCB congeners, nor did the study measure exposure to dioxins (Shin et al. 2010). It remains unclear whether this association would also be seen in a population-based study with more complete control for confounders and whether exposure to dioxins is also associated with longer LTL.

Although no prior epidemiologic study has examined the effects of dioxin exposure on LTL, some preliminary *in vitro* findings suggest a possible role for telomerase and telomere length in dioxin-mediated carcinogenesis. In one *in vitro* study using human choriocarcinoma cells, TERT expression (and therefore telomerase) was up-regulated in cells treated with dioxin (Sarkar et al. 2006). Because dioxins are potent aryl hydrocarbon receptor (AhR) agonists (Chopra and Schrenk 2011), the authors of that study suggested that telomerase up-regulation may be mediated via AhR activation (Sarkar et al. 2006). Activated telomerase, which elongates telomeres, is thought to confer uncontrolled replicative ability on a cell (Ruden and Puri 2013). Additionally, dioxins have been shown to immortalize human keratinocytes and to repress transcription of tumor suppressor genes p53 and p16^INK4a^ *in vitro* (Ray and Swanson 2004).

In this analysis, we conducted a cross-sectional study to examine the association between exposure to PCBs, dioxins, and furans and LTL, using a representative sample of U.S. adults from NHANES 2001–2002. We hypothesized that exposure to dioxins, furans, and PCBs would be associated with longer LTL. Additionally, we hypothesized that the association would be stronger for congeners with greater AhR affinity.

## Methods

We used data from the 2001–2002 cycle of the National Health and Nutrition Examination Survey (NHANES) to examine the association between lipid-adjusted serum measurements of POPs (34 PCBs, 7 dioxins, and 9 furans) and relative LTL. NHANES is an ongoing series of cross-sectional national surveys conducted by the U.S. Centers for Disease Control and Prevention (CDC), including both physical examinations and questionnaires. NHANES uses probability-based methods to obtain a sample that is representative of the noninstitutionalized population of the United States. All participants provide written informed consent, and the National Center for Health Statistics obtains institutional review board approval to conduct the surveys (Zipf et al. 2013).

### Study Population

In the 2001–2002 cycle, 11,039 people were interviewed by NHANES. Stored samples were available and sufficient to estimate telomere length for 4,260 participants ≥ 20 years of age who provided a blood sample and consented to the use of their DNA (78.7% of all interviewed participants ≥ 20 years of age). Women, individuals below the poverty level, individuals with at least a high school education, non-Hispanic blacks, and individuals over age 60 were less likely to consent to use of their DNA samples (McQuillan et al. 2006). From the population with sufficient DNA samples to generate LTL data, we then excluded individuals without environmental chemical analysis data (*n* = 2,850) or who were missing data on body mass index (BMI) (*n* = 70), education (*n* = 2), or serum cotinine (*n* = 8), leaving a study population of 1,330 individuals (see Supplemental Material, Figure S1).

### Exposure Assessment (PCB/Dioxin Measurements)

Serum specimens were spiked with ^13^C_12_-labeled internal standards, and the dioxin or PCB congener was isolated in hexane using a C18 solid phase extraction. To isolate PCBs, samples were then extracted through neutral silica and Florosil SPE columns (CDC 2002a); to isolate dioxins, samples underwent Power-Prep/6 (Fluid Management Systems) automated cleanup and enrichment procedures using multi-layered silica gel and alumina columns coupled to an AX-21 carbon column (CDC 2002b). Dioxins were isolated in reverse direction from carbon using toluene (CDC 2002b). Each analytical run of PCBs, dioxins, or furans was blinded and included blanks and quality-control samples. Before quantifying the PCB, dioxin, or furan in a sample, the sample was reconstituted with ^13^C_12_-labeled external standard. PCBs, dioxins, and furans were then measured using high-resolution gas chromatography/isotope-dilution high-resolution mass spectrometry (CDC 2002a, 2002b). Limits of detection (LOD) were reported for every sample. Samples with greater serum volume had lower detection limits (CDC 2002a, 2002b). Typically, detection limits were ~2 ng/g, although they could be as high as 10.5 ng/g (the LOD range for each chemical is reported in Table 1). The CDC replaced concentrations below the LOD with the sample-specific LOD divided by the square root of 2. Coefficients of variation ranged from 7.7 to 28 for the PCBs and from 5.3 to 15.3 for the dioxins and furans, varying by congener and sample lot (CDC 2002a, 2002b). Serum lipids were calculated using an enzymatic summation method (Akins et al. 1989).

**Table 1:** LOD and weights used in metrics.

Pollutant	*n*	LOD range^*a,b*^	> LOD (%)	Non–dioxin-like PCBs^*b*^	Non-ortho PCBs^*b*^	TEQ^*b*^
PCBs
PCB-74	1,323	1.6–10.5	67.7	1.0		
PCB-99	1,301	< 2.8–10.5	62.6	1.0		
PCB-105	1,323	1.6–10.5	21.3			0.00003
PCB-118	1,323	2.8–10.5	73.1			0.00003
PCB-126	1,137	1.8–10.5	88.0		1.0	0.1
PCB-138	1,319	3.0–10.5	93.3	1.0		
PCB-153	1,323	3.0–10.5	96.3	1.0		
PCB-156	1,317	1.6–10.5	54.0			0.00003
PCB-157	1,316	0.8–10.5	7.5			0.00003
PCB-167	1,317	< 1.3–10.5	10.8			0.00003
PCB-169	1,135	2.4–10.5	88.1		1.0	0.03
PCB-170	1,319	< 2.8–10.5	75.1	1.0		
PCB-180	1,322	2.8–10.5	89.4	1.0		
PCB-187	1,323	1.6–10.5	66.1	1.0		
PCB-189	1,320	0.6–10.5	0.23			0.00003
PCB-194	1,309	1.6–10.5	60.9	1.0		
PCB-196	1,318	1.6–10.5	54.8	1.0		
PCB-199	1,311	1.6–10.5	58.4	1.0		
Dioxins
2,3,7,8-TCDD	1,137	0.4–5.7	11.7			1.0
1,2,3,7,8-PeCDD	1,142	1.0–5.9	33.1			1.0
1,2,3,4,7,8-HxCDD	1,145	1.3–8.9	33.7			0.1
1,2,3,6,7,8- HxCDD	1,140	2.0–8.6	92.7			0.1
1,2,3,7,8,9- HxCDD	1,144	1.3–9.3	40.4			0.1
1,2,3,4,6,7,8-HpCDD	1,128	2.7–9.6	98.6			0.01
1,2,3,4,6,7,8,9-OCDD	1,083	52.0–318.8	80.8			0.0003
Furans
2,3,7,8-TCDF	1,137	0.4–5.2	0.8			0.1
1,2,3,7,8-PeCDF	1,141	0.4–5.8	0.7			0.03
2,3,4,7,8- PeCDF	1,137	1.0–5.8	64.5			0.3
1,2,3,4,7,8-HxCDF	1,132	1.4–6.4	81.1			0.1
1,2,3,6,7,8- HxCDF	1,143	1.1–6.1	68.3			0.1
1,2,3,7,8,9- HxCDF	1,130	0.6–5.9	0.1			0.1
2,3,4,6,7,8- HxCDF	1,136	1.0–5.8	11.0			0.1
1,2,3,4,6,7,8-HpCDF	1,126	1.3–6.9	89.4			0.01
1,2,3,4,7,8,9-HpCDF	1,131	0.7–6.9	0.1			0.01
Abbreviations: HpCDD, heptachlorodibenzo-*p*-dioxin; HpCDF, heptachlorodibenzofuran; HxCDD, hexachlorodibenzo-*p*-dioxin; HxCDF, hexachlorodibenzofuran; LOD, limit of detection; OCDD, octachlorodibenzo-*p*-dioxin; PCB, polychlorinated biphenyl; PeCDD, pentachlorodibenzo-*p*-dioxin; PeCDF, pentachlorodibenzofuran; TCDD, tetrachlorodibenzo-*p*-dioxin; TCDF, tetrachlorodibenzofuran; TEQ, toxic equivalent.^***a***^LODs are represented as a range because they vary by sample volume, which differed among participants. ^***b***^Units are picograms per gram lipid for PCB-126, PCB-169, dioxins, and furans; and nanograms per gram lipid for other PCBs.						

### Telomere Length Measurement

Aliquots of purified DNA were provided by the laboratory at the Division of Health and Nutrition Examination Surveys, National Center for Health Statistics, Centers for Disease Control and Prevention. DNA was extracted from whole blood using the Puregene (D-50K) kit protocol (Gentra Systems, Inc., Minneapolis, Minnesota) and stored at –80° C. The telomere length assay was performed in the laboratory of E. Blackburn (University of California, San Francisco), using the quantitative polymerase chain reaction (qPCR) method to measure telomere length relative to standard reference DNA (T/S ratio) (Cawthon 2002; Lin et al. 2009). The telomere thermal cycling profile consisted of cycling for T (telomic) PCR: 96°C for 1 min; denature at 96°C for 1 sec, anneal/extend at 54°C for 60 sec, with fluorescence data collection, 30 cycles. Cycling for S (single copy gene) PCR consisted of the following: 96°C for 1 min; denature at 95°C for 15 sec, anneal at 58°C for 1 sec, extend at 72°C for 20 sec, 8 cycles; followed by denature at 96°C for 1 sec, anneal at 58°C for 1 sec, extend at 72°C for 20 sec, hold at 83°C for 5 sec with data collection, 35 cycles.

The primers for the telomere PCR were *tel1b* [5´-CGG​TTT​(GTTTGG)_5_​GTT-3´], used at a final concentration of 100 nM, and *tel2b* [5´-GGC​TTG​(CCTTAC)_5_​CCT-3´], used at a final concentration of 900 nM. The primers for the single-copy gene (human beta-globin) PCR were *hbg1* (5´ GCT​TCT​GAC​ACA​ACT​GTG​TTC​ACT​AGC-3´), used at a final concentration of 300 nM, and *hbg2* (5´-CAC​CAA​CTT​CAT​CCA​CGT​TCACC-3´), used at a final concentration of 700 nM. The final reaction mix contained 20 mM Tris-hydrochloride (HCl), pH 8.4; 50 mM potassium chloride (KCl); 200 μM each deoxynucleotide (dNTP); 1% dimethyl sulfoxide (DMSO); 0.4× SYBR Green I; 22 ng *Escherichia coli* DNA per reaction; and 0.4 units of Platinum Taq DNA polymerase (Invitrogen Inc.) per 11-μL reaction.

Each sample was assayed three times in duplicate wells, producing six data points. The mean T/S ratio values were calculated, and the T/S ratio that differed most from the mean for the group of replicates was marked as a potential outlier. The mean was calculated a second time, excluding the potential outlier. The potential outlier was determined to be a true outlier if the absolute value of the log of the ratio between the recalculated mean (excluding the potential outlier) and the value of the potential outlier was greater than 0.4 (98.7% of all samples contained no outliers) (Needham et al. 2013). DNA samples were coded, and the lab was blinded to all other measurements in the study. The CDC conducted a quality control review before linking the telomere data to the NHANES 1999–2002 public use data files.

### Statistical Analysis

Based on *in vivo* evidence of toxicity, the World Health Organization (WHO) assigned each congener a toxic equivalency factor (TEF), a measure of relative potency compared with that of reference chemical 2,3,7,8-tetrachlorodibenzo-*p*-dioxin (Van den Berg et al. 2006). Because the TEF is partially based on AhR affinity, we used each congener’s TEF (according to the WHO 2005 summary) as a proxy for affinity for the AhR in order to group congeners by expected effect (Van den Berg et al. 2006).

After excluding congeners detected in 0% of samples (PCB-81 and 1,2,3,4,6,7,8,9-octachlorodibenzofuran) and PCBs without AhR affinity that were detected in less than 50% of samples (PCBs 52, 66, 87, 101, 110, 128, 146, 149, 151, 172, 177, 178, 183, 195, and 206), we created three summary metrics with varying TEFs from lipid-adjusted serum measurements of the POPs: non–dioxin-like PCBs, non-ortho PCBs, and toxic equivalent (TEQ). Before summing, all pollutant concentrations were converted to picograms per gram lipid to account for individual differences in serum lipids because dioxins distribute to the lipid fraction of blood (Schisterman et al. 2005).

The non–dioxin-like PCB metric was created by summing the lipid-corrected concentrations of 10 PCBs with no TEFs and no AhR affinity (PCBs 74, 99, 138, 153, 170, 180, 187, 194, 196, and 199; TEF value range 0.0–0.0) (Table 1) (Van den Berg et al. 2006). The non-ortho PCB metric was the sum of the lipid-corrected concentrations of 2 non–ortho-substituted PCBs, which had high TEFs and high AhR affinity (PCBs 126 and 169; TEF value range 0.03–0.10) (Table 1) (Van den Berg et al. 2006). The TEQ metric was the sum of the lipid-corrected concentrations of 7 chlorinated dibenzo-*p*-dioxins, 9 dibenzofurans, 2 non–ortho-substituted PCBs, and 6 mono–ortho-substituted PCBs, weighted using TEFs from the WHO 2005 summary in the following manner: TEQ = Σ[(congener’s TEF) × (congener’s concentration)] (TEQ value range 0.0003–1.0) (Table 1) (Van den Berg et al. 2006). Although the non–ortho-substituted PCBs were included in both the non-ortho PCB metric and the TEQ metric, the non-ortho PCB metric was created without weighting, whereas the TEQ was weighted using TEF values. Only participants with complete data on all congeners in a metric were included in the metric. All metrics were natural log–transformed to account for their nonnormal distributions.

To estimate the association between pollutants and LTL, the metrics were modeled continuously and in quartiles. Quartiles were calculated using a weighted distribution based on NHANES population weights. Additionally, a test for trend was performed by modeling the integer value of each quartile (i.e., 0, 1, 2, 3) as an ordinal term and using its *p*-value as a test of departures from the null hypothesis of no linear trend.

We used multivariable linear regression models to assess the relationship between serum levels of POPs and relative LTL. Covariates were selected *a priori* based on factors shown to be associated with LTL in this population (Needham et al. 2013; Zota et al. 2015). Two multivariable models were used. The first model was adjusted for age (continuous) and age^2^ (continuous). The second model was adjusted for age (continuous), age^2^ (continuous), sex, race/ethnicity (non-Hispanic white, non-Hispanic black, Mexican American, other), educational attainment (less than high school, high school graduate, some college, college or more) BMI (< 25, 25–29.9, ≥ 30), smoking (natural log–transformed cotinine), and blood cell count and distribution (white blood cell count, percent lymphocytes, percent monocytes, percent neutrophils, percent eosinophils, percent basophils). Blood cell count and distribution variables were included because LTL is measured in immune cells and because blood cell count and distribution are associated with serum PCBs (Serdar et al. 2014). We estimated the percent difference in LTL for a doubling of exposure concentration as [*e* ^(ln2 × β)^ – 1] × 100%, with the 95% confidence inverval (CI) estimated as {*e* ^[ln2 × (β ± 2.131 × SE)]^ – 1} × 100%. For quartiles, percent differences were calculated using the formula {[*e* ^(β)^ – 1] × 100%}, with 95% CIs estimated as {[*e* ^(β ± 2.131 × SE)^ – 1] × 100}, where β and SE are the estimated regression coefficient and the standard error, respectively. Percent differences were estimated by comparing each of the upper three quartiles with the lowest quartile, and tests for linear trends were conducted by modeling quartiles as an ordinal variable. The degrees of freedom were calculated by subtracting the number of clusters in the first level of sampling (strata) from the number of clusters [primary sampling units (PSUs)] in the second level of sampling [CDC/National Center for Health Statistics (NCHS) 2011]. Our study sample had 15 degrees of freedom, so we used a critical value of ±2.131 from the *t* distribution for the calculation of confidence intervals. All analyses were adjusted for the sampling design of the NHANES survey using the 2-year dioxin subsample weights. A (two-sided) *p*-value < 0.05 was considered statistically significant.

To further differentiate the effects of congeners with AhR affinity from those without AhR affinity, we included a second exposure metric in the final, adjusted multivariable models of each exposure. Specifically, models of association of non–dioxin-like PCBs were additionally adjusted for non-ortho PCBs, and models of non-ortho PCBs and TEQ were additionally adjusted for non–dioxin-like PCBs. These adjustments were made because the non–dioxin-like metric is highly correlated with both the non-ortho PCBs and TEQ (Pearson correlations of 0.77 and 0.79, respectively, both *p* < 0.01). Therefore, the non–dioxin-like PCBs may confound the association between the other metrics and LTL; similarly, the other metrics may confound the association between non–dioxin-like PCBs and LTL. Because the metrics have different AhR affinities, including the second metric with different AhR affinity controlled for this potential confounding. We also used these multivariate models to test for interaction by age, sex, race/ethnicity, and cancer diagnosis. In these models, age was categorized into three groups (20–39, 40–59, ≥ 60), and race/ethnicity was categorized into four groups as described above. Interactions were assessed for statistical significance using the *p*-value associated with the *F* statistic for the product term created by multiplying each categorical covariate by the natural log–transformed continuous exposure metric. Participants answering, “Yes,” to the question, “Have you ever been told by a doctor or other health care professional that you had cancer or a malignancy of any kind?” were recorded as having a cancer diagnosis.

In addition to the main analyses, we conducted several sensitivity analyses to ensure that the values below the LOD, which constituted the majority of the data for some congeners, did not affect the overall results. We modeled TEQ in two additional ways: first, we included only congeners that were detected in > 50% of the samples (*n* = 11); second, we included only congeners that were detected in > 33% of the samples (*n* = 14). We also used multiple imputation to generate values for all observations that were below the LOD in each congener, assuming that the values fell between zero and LOD divided by the square root of 2 for each congener and that the congeners were log-normally distributed. We averaged the results of five runs of the lifereg model in SAS v.9.3 (SAS Institute Inc., Cary, NC) to generate the imputed values. Only congeners with ≥ 10% of values detected were imputed to ensure valid imputation; for congeners with < 10% of values detected, the sample-specific LOD divided by the square root of 2 was used for < LOD values.

A second sensitivity analysis was performed to assess the effects of lipid adjustment because dividing serum POPs by serum lipids could introduce bias if the serum lipids were associated with LTL (Schisterman et al. 2005). Because NHANES 2001–2002 does not provide lipid concentrations as a separate variable, serum lipids were calculated by dividing each congener’s lipid-adjusted value by its wet-weight value for all values above the LOD. The final serum lipid value assigned to each participant was the average of the lipids calculated from PCB-74, PCB-138, PCB-153, PCB-180, and 1,2,3,4,6,7,8-heptachlorodibenzo-*p*-dioxin ratios. Fifteen participants had no measured congeners above the LOD, and their lipid levels were marked as missing. Multivariable models were run using congener wet-weight values, including serum lipids as a separate variable in the model.

## Results

Eighteen of the 34 PCBs (53%) were eligible for inclusion in the summed exposure metrics (Table 1). LTL was positively associated with serum cotinine and inversely associated with age (Table 2). Participants with < high school education had shorter LTL than those with more education; participants with BMI < 25 had longer LTL than those with higher BMIs (Table 2). LTL was shorter in white participants and longer in Mexican American participants, and it was shorter in people with a cancer diagnosis (Table 2). Levels of non–dioxin-like PCBs, non-ortho PCBs, and TEQ were significantly positively associated with age, and levels of non-ortho PCBs and TEQ were significantly negatively associated with serum cotinine (Table 3). Mexican American participants had the lowest levels of non–dioxin-like PCBs, non-ortho PCBs, and TEQ (Table 3). Participants with a cancer diagnosis had higher levels of PCBs, non-ortho PCBs, and TEQ (Table 3).

**Table 2:** Demographic characteristics and leukocyte telomere length (T/S ratio × 100) in the study population drawn from NHANES 2001–2002. All analyses were adjusted for NHANES sample weights.

Covariate	Participants (*n* = 1,330)% (SE)	LTL(T/S ratio × 100)geometric mean (95% CI)^*a*^
Whole population		106.6 (105.5, 107.6)
Serum cotinine (ng/mL)
< 0.015	19.0 (2.9)	104.1 (101.8, 106.4)**
0.015–9.90	50.9 (2.7)	106.0 (104.9, 107.2)
≥ 9.91	30.1 (1.6)	109.1 (108.0, 110.2)
Age (years)
20–39	42.3 (2.4)	117.8 (112.2, 123.4)**
40–59	38.2 (1.7)	107.2 (101.6, 112.7)
≥ 60	19.5 (1.5)	92.9 (89.3, 96.5)
Sex
Male	49.3 (1.6)	106.8 (105.4, 108.2)
Female	50.7 (1.6)	106.3 (105.4, 107.2)
Race/ethnicity
Non-Hispanic white	73.3 (2.9)	105.5 (104.2, 106.8)**
Non-Hispanic black	10.3 (2.1)	108.5 (107.2, 109.8)
Mexican American	7.0 (0.8)	111.3 (110.6, 112.0)
Other	9.4 (2.0)	109.3 (107.7, 111.0)
Education
< High school	19.8 (1.5)	105.4 (103.6, 107.2)*
High school graduate	24.3 (1.1)	106.8 (104.7, 108.8)
Some college	30.4 (2.1)	107.6 (106.2, 109.1)
≥ College graduate	25.5 (1.8)	106.0 (104.9, 107.1)
BMI (kg/m^2^)
< 25	34.8 (0.9)	108.3 (107.3, 109.2)**
25–29.9	35.6 (1.4)	105.7 (104.3, 107.1)
≥ 30	29.7 (1.6)	105.6 (104.1, 107.1)
Cancer diagnosis^*b*^
No	91.2 (1.1)	107.4 (106.4, 108.4)**
Yes	8.8 (1.1)	97.6 (95.0, 100.2)
Abbreviations: BMI, body mass index; CI, confidence interval; LTL, leukocyte telomere length; NHANES, National Health and Nutrition Examination Survey; SE, standard error.^***a***^LTL is adjusted for continuous age, except for the LTL values in the age group categories. ^***b***^Two participants were missing data on cancer diagnosis (*n* = 1,328). Participants answering “Yes” to the question, “Have you ever been told by a doctor or other health care professional that you had cancer or a malignancy of any kind?” were recorded as having a cancer diagnosis. *p*-Values were calculated using analysis of variance (ANOVA) tests within each demographic category. *p < 0.05. **p < 0.01.		

**Table 3:** Lipid-adjusted serum pollutant levels by summed metric in NHANES 2001–2002.

Variable	Non–dioxin-like PCBs(ng/g lipid) (*n* = 1,273)	Non-ortho PCBs(pg/g lipid) (*n* = 1,135)	TEQ(pg/g lipid)(*n* = 1,003)
Whole population	136.9 (125.4, 149.4)	43.3 (39.7, 47.3)	18.6 (17.1, 20.2)
Serum cotinine (ng/mL)
< 0.015	154.0 (125.8, 188.4)	50.5 (43.4, 58.8)**	20.2 (17.7, 23.1)**
0.015–9.9	137.6 (125.8, 150.4)	46.7 (42.9, 50.8)	19.4 (17.7, 21.2)
≥ 9.91	125.7 (113.0, 139.7)	34.3 (29.8, 39.5)	16.4 (14.8, 18.3)
Age (years)
20–39	76.6 (70.5, 83.1)**	27.7 (24.8, 31.0)**	13.6 (12.7, 14.7)**
40–59	171.4 (156.7, 187.6)	49.3 (45.7, 53.2)	19.3 (17.7, 20.9)
≥ 60	309.4 (294.0, 325.5)	84.7 (78.4, 91.5)	32.8 (29.5, 36.6)
Sex
Male	137.8 (123.4, 153.8)	43.5 (39.2, 48.4)	18.3 (16.9, 19.9)
Female	136.1 (126.0, 147.0)	43.2 (39.6, 47.0)	18.8 (17.1, 20.6)
Race/ethnicity
Non-Hispanic white	147.2 (133.4, 162.4)**	45.5 (41.4, 50.0)**	19.3 (17.6, 21.1)**
Non-Hispanic black	157.4 (142.0, 174.4)	42.9 (38.4, 48.1)	20.4 (18.7, 22.2)
Mexican American	71.3 (67.4, 75.5)	29.3 (26.9, 31.9)	14.0 (13.4, 14.5)
Other	109.4 (88.6, 135.2)	40.6 (32.2, 51.2)	16.1 (14.0, 18.4)
Education
< High school	136.9 (117.5, 159.6)	40.9 (35.2, 47.4)	19.4 (16.9, 22.2)
High school graduate	133.9 (116.9, 153.3)	43.3 (39.4, 47.5)	19.4 (17.4, 21.6)
Some college	130.4 (117.4, 145.0)	42.7 (38.0, 48.0)	17.9 (16.6, 19.3)
≥ College graduate	148.1 (129.8, 169.1)	46.2 (38.9, 54.9)	17.9 (16.2, 19.8)
BMI (kg/m^2^)
< 25	140.5 (126.6, 155.9)	42.1 (38.6, 45.9)	18.9 (17.6, 20.4)
25–29.9	138.6 (125.3, 153.4)	42.5 (37.3, 48.3)	17.9 (16.1, 19.9)
≥ 30	130.9 (114.2, 149.9)	45.9 (40.9, 51.6)	19.1 (17.1, 21.4)
Cancer diagnosis^*a*^
Yes	208.1 (184.3, 235.0)**	60.2 (51.0, 71.1)**	25.3 (21.6, 29.7)**
No	131.7 (121.1, 143.1)	41.9 (38.5, 45.6)	18.0 (16.7, 19.4)
Abbreviations: BMI, body mass index; NHANES, National Health and Nutrition Examination Survey; PCB, polychlorinated biphenyl; TEQ, toxic equivalent. *p*-Values were calculated using analysis of variance (ANOVA) tests within each demographic category. Reported values are geometric means and 95% confidence intervals. All analyses were adjusted for NHANES sample weights.^***a***^Two participants were missing data on cancer diagnosis. Participants answering “Yes” to the question, “Have you ever been told by a doctor or other health care professional that you had cancer or a malignancy of any kind?” were recorded as having a cancer diagnosis. **p < 0.01.			

Exposure to non-ortho PCBs was associated with longer LTL. Each doubling of the non-ortho PCB metric was associated with 3.74% (95% CI: 2.10, 5.40) longer LTL in fully adjusted models (Table 4, Model 3). In quartile models, the highest (vs. lowest) quartile of exposure was associated with 9.16% (95% CI: 2.96, 15.73) longer LTL (*p* for trend = 0.0036, Table 4, Model 3). The association was slightly stronger in models without adjustment for non–dioxin-like PCBs (Table 4, Model 2). In those models, each doubling of non–ortho-PCBs was associated with 4.31% (95% CI: 2.76, 5.88) longer LTL (*p* for trend = 0.0011, Table 4, Model 2).

**Table 4:** Percent difference (95% CI) in leukocyte telomere length (T/S ratio) by non–dioxin-like PCBs, non-ortho PCBs, and the toxic equivalent (TEQ).

Metric	*n*	Model 1^*a*^ percent difference (95% CI)	*n*	Model 2^*b*^ percent difference (95% CI)	*n*	Model 3^*c*^ percent difference (95% CI)
Non–dioxin-like PCBs
Per doubling of exposure	1,273	4.09 (1.16, 7.11)**	1,267	3.80 (0.80, 6.90)*	1,094	0.42 (–2.39, 3.32)
Quartile 1 (≤ 74.6 ng/g)		Reference		Reference		Reference
Quartile 2 (74.7–135.9 ng/g)		3.50 (–3.13, 10.58)		3.04 (–3.19, 9.67)		0.53 (–4.96, 6.33)
Quartile 3 (136.0–245.2 ng/g)		7.68 (1.52, 14.22)*		7.20 (1.49, 13.23)*		1.21 (–3.75, 6.43)
Quartile 4 (> 245.2 ng/g)		10.55 (2.07, 19.74)*		9.40 (0.88, 18.64)*		0.84 (–5.37, 7.45)
*p *trend		0.0091**		0.019*		0.74
Non-ortho PCBs
Per doubling of exposure	1,135	4.26 (2.42, 6.14)**	1,129	4.31 (2.76, 5.88)**	1,094	3.74 (2.10, 5.40)**
Quartile 1 (≤ 26.8 pg/g)		Reference		Reference		Reference
Quartile 2 (26.9–44.7 pg/g)		0.94 (–3.90, 6.17)		0.85 (–3.96, 5.89)		1.30 (–3.66, 6.53)
Quartile 3 (44.8–71.5 pg/g)		6.45 (1.80, 11.30)**		6.17 (2.15, 10.35)**		5.21 (0.92, 9.68)*
Quartile 4 (> 71.5 pg/g)		10.60 (3.29, 18.44)**		10.63 (3.98, 17.71)**		9.16 (2.96, 15.73)**
*p *trend		0.0029**		0.0011**		0.0036**
TEQ
Per doubling of exposure	1,003	5.59 (1.99, 9.31)**	999	5.76 (2.27, 9.37)**	976	5.29 (1.66, 9.05)**
Quartile 1 (≤ 12.7 pg/g)		Reference		Reference		Reference
Quartile 2 (12.8–17.3 pg/g)		1.45 (–3.06, 6.17)		2.05 (–2.71, 7.05)		1.43 (–3.41, 6.52)
Quartile 3 (17.4–25.3 pg/g)		3.71 (–3.63, 11.60)		4.11 (–3.11, 11.88)		3.25 (–4.04, 11.10)
Quartile 4 (> 25.3 pg/g)		9.37 (1.55, 17.79)*		9.23 (1.28, 17.80)*		7.84 (–0.53, 16.92)
*p *trend		0.048*		0.051		0.11
Abbreviations: CI, confidence interval; NHANES, National Health and Nutrition Examination Survey; PCB, polychlorinated biphenyl. All analyses were adjusted for NHANES sample weights.^***a***^Model 1 is adjusted for age, age^2^. ^***b***^Model 2 is adjusted for age, age^2^, sex, race/ethnicity, BMI, log(cotinine), white blood cell count, percent lymphocytes, percent monocytes, percent neutrophils, percent eosinophils, percent basophils. ^***c***^Model 3 is additionally adjusted for a second exposure metric. Non–dioxin-like PCBs are adjusted for non-ortho PCBs. Non-ortho PCBs and TEQ are adjusted for non–dioxin-like PCBs. *p < 0.05. **p < 0.01.						

Exposure to TEQ was also associated with longer LTL. Each doubling of the TEQ metric was associated with 5.29% (95% CI: 1.66, 9.05) longer LTL in fully adjusted models (Table 4, Model 3). In quartile models, the association was not significant. The highest (vs. lowest) quartile of exposure was associated with 7.84% (95% CI: –0.53, 16.92) longer LTL (*p* for trend = 0.11, Table 4, Model 3). The association was slightly stronger in models without adjustment for non–dioxin-like PCBs (Table 4, Model 2). In those models, each doubling of TEQ was associated with 5.76% (95% CI: 2.27, 9.37) longer LTL (*p* for trend = 0.051, Table 4, Model 2).

Exposure to non–dioxin-like PCBs was not associated with longer LTL after adjustment for non-ortho PCBs. In fully adjusted models, each doubling of the non–dioxin-like PCBs metric was associated with 0.42% (95% CI: –2.39, 3.32) longer LTL (Table 4, Model 3). Quartiles of non–dioxin-like PCBs were also not associated with LTL (Table 4, Model 3). However, in models that were not adjusted for non-ortho PCBs, each doubling of non–dioxin-like PCBs was associated with 3.80% (95% CI: 0.80, 6.90) longer LTL, and in quartile models, the highest (vs. lowest) quartile of exposure was associated with 9.40% (95% CI: 0.88, 18.64) longer LTL (*p* for trend = 0.019, Table 4, Model 2).

**Figure 1:**
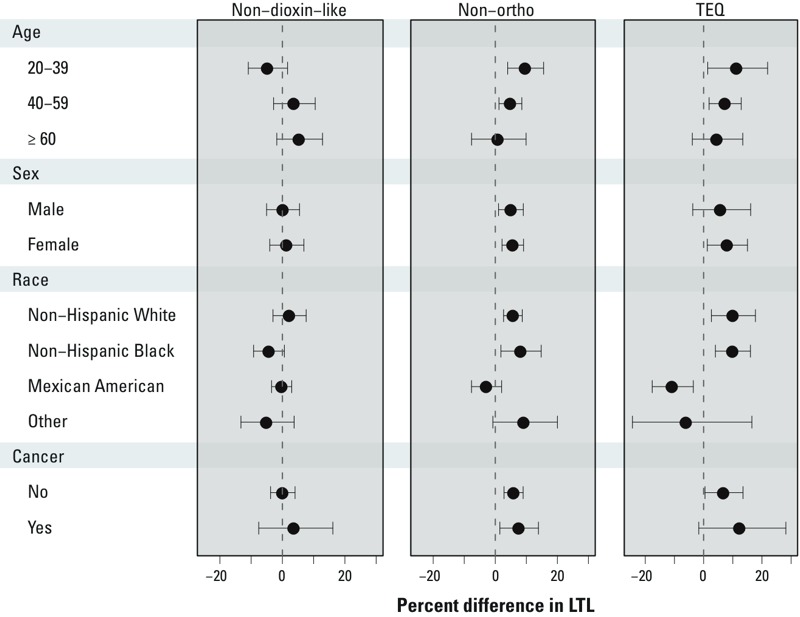
Association between persistent organic pollutants (POPs) metric exposure and leukocyte telomere length (LTL) in the general U.S. population. Percent difference estimates were generated from stratified models. *p*-Values were derived from linear regression models with multiplicative interaction terms for age [non–dioxin-like *p*_interaction_ = 0.14; non-ortho *p*_interaction_ = 0.80; toxic equivalent (TEQ) *p*_interaction_ = 0.87]; sex (non–dioxin-like *p*_interaction_ = 0.18; non-ortho *p*_interaction_ = 0.23; TEQ *p*_interaction_ = 0.13); race (non–dioxin-like *p*_interaction_ = 0.21; non-ortho *p*_interaction_ = 0.19; TEQ *p*_interaction_ < 0.01); and cancer status (non–dioxin-like *p*_interaction_ = 0.72; non-ortho *p*_interaction_ = 0.68; TEQ *p*_interaction_ = 0.37). Regression models were adjusted for age, age^2^, sex, race/ethnicity, body mass index (BMI), log(cotinine), white blood cell count, percent lymphocytes, percent monocytes, percent neutrophils, percent eosinophils, percent basophils. Non–dioxin-like polychorinated biphenyls (PCBs) were adjusted for non-ortho PCBs. Non-ortho PCBs and TEQ were adjusted for non–dioxin-like PCBs. Data points represent percent difference in LTL per each log-unit increase in the exposure metric, and error bars represent 95% confidence intervals.

Tests for interaction by age, sex, and cancer diagnosis were not significant (Figure 1). Tests for interaction by race/ethnicity were not significant for non–dioxin-like PCBs or non-ortho PCBs but were significant for TEQ (*p* < 0.01, Figure 1). Exposure to TEQ was associated with significantly longer LTL among non-Hispanic whites and non-Hispanic blacks, but it was associated with significantly shorter LTL among Mexican Americans (Figure 1). Figure 1 *p*-values were derived from linear regression models with multiplicative interaction terms; percent difference estimates were generated from stratified models.

Sensitivity analyses examining the effects of the treatment of data below the LOD on the overall results did not meaningfully alter the findings (see Supplemental Material, Table S1). Models based on congener wet weights, adjusting for serum lipids as a separate covariate, largely showed the same pattern of results as the models that used lipid-adjusted values (see Supplemental Material, Table S2).

## Discussion

In this analysis, exposure to non-ortho PCBs and TEQ was associated with longer LTL even after adjustment for non–dioxin-like PCB exposure. Exposure to non–dioxin-like PCBs was no longer associated with LTL after adjustment for non-ortho PCB exposure. Each doubling of exposure to non-ortho PCBs or TEQ was associated with 3–6% longer LTL in fully adjusted models (*p* < 0.01 for both exposures). Non-ortho PCBs were also associated with longer LTL in quartile models, with evidence of positive dose–response (*p* for trend = 0.0036). TEQ quartile models were suggestive of positive, monotonic dose–response, but after adjustment for non–dioxin-like PCBs, the trend failed to reach significance (*p* for trend = 0.11) despite the significant association in continuous models.

Our results suggest that the strength of the association varies by congener TEF. Exposure to non-ortho PCBs and TEQ (with high TEFs and AhR affinity) were associated with longer LTL in linear models even after adjustment for exposure to non–dioxin-like PCBs, which have no AhR affinity. However, non–dioxin-like PCBs were not associated with LTL after adjustment for non-ortho PCBs. These results align well with those of a previous epidemiological study that investigated this association in PCBs (Shin et al. 2010) as well as with preliminary *in vitro* data suggesting that AhR activation can activate telomerase, thereby elongating LTL (Sarkar et al. 2006).

Although both non-ortho PCBs and TEQ were associated with longer LTL in linear models, the positive dose–response relationship was weaker for TEQ when exposure was modeled as quartiles. The TEQ metric incorporated congeners with very strong as well as very weak TEFs and AhR affinity, and this heterogeneity may have increased the error when estimating the effect of the metric. Alternatively, the pattern of results may be due to the greater prevalence of undetected values in the TEQ metric, which would be expected to introduce random error, especially in the lower quartiles. The potential also exists for mutual confounding between the two metrics; this was not tested in the present analysis.

The relationship between LTL and cancer is complicated, varying by cancer type and sometimes by demographic characteristics, such as age of the cancer patient or duration between LTL measurement and cancer diagnosis (Boardman et al. 2014; Hou et al. 2015). In general, shorter LTL appears to increase cancer risk (Willeit et al. 2010). However, many cancers linked to dioxin exposure have been associated with longer LTL in multiple epidemiological studies; these cancers include lung cancer (Sanchez-Espiridion et al. 2014; Seow et al. 2014), soft tissue sarcoma (Xie et al. 2013), and non-Hodgkin lymphoma (Lan et al. 2009). In fact, among 20 published studies investigating the association between PCB/dioxin-related cancer risk and LTL, 15 reported positive associations, largely based on tertile or quartile trend tests (see Supplemental Material, Table S3). The role of telomere biology in dioxin carcinogenesis warrants further research in both experimental and observational studies.

To explore the possible connections among dioxins, LTL, and cancer in this population, we tested for effect modification by cancer diagnosis. However, the statistical interaction was not significant, and the associations were comparable between those with a previous cancer diagnosis and those without a diagnosis. Our analysis by cancer diagnosis was limited by the small number of participants with a cancer diagnosis, the lack of information on clinical characteristics of the cancer cases, an inability to divide the analysis by cancer type, potential clinical heterogeneity among the cancer cases, and variability in time since cancer diagnosis. The relationship between POPs and LTL in cancer patients should be examined in future studies.

This study had several important strengths. Because we used NHANES data, we were able to examine these associations in a large population. Additionally, NHANES collected precise data on serum concentrations of 51 PCBs, dioxins, and furans, which allowed us to accurately assess each participant’s exposure to a range of relevant POPs, to group congeners according to shared biochemical characteristics, and to control for simultaneous exposure to multiple congeners. Finally, we were able to control for multiple possible demographic and chemical confounders, including blood cell count and distribution.

However, this study was also subject to limitations. The cross-sectional design of NHANES prevents us from making causal inference or establishing a temporal relationship between PCB, dioxin, or furan exposure and LTL. Additionally, many of the dioxins and furans measured by NHANES were detected in very low levels in most participants, limiting the number of observations above the LOD and thereby reducing precision in the lower TEQ quartiles. We did not adjust for missingness probabilities in the data, so our sample may have been biased because participants who did not consent to use of their DNA were nonrandom. Finally, the strong association between age and serum levels of each pollutant likely reflects two different age-varying factors: *a*) much higher exposures historically (before these chemicals were banned); and *b*) accumulation and persistence of these chemicals in the human body over time. Older participants were therefore exposed at a higher level, for a longer period of time, than younger participants. Our findings may have been affected by our inability to fully model these age-specific effects despite our inclusion of age (and age^2^) terms in the regression models.

## Conclusions

Our analysis shows that exposure to non-ortho PCBs and TEQ was associated with increased LTL in our study population of U.S. adults, contributing population-level findings to the evidence that exposure to environmental contaminants may influence telomere regulation. This association was not seen for exposure to non–dioxin-like PCBs, suggesting that the pattern of results may be driven by the congeners’ varying relative potency and affinity for the AhR. Because many dioxin-associated cancers are also associated with increased LTL, these results may provide insight into the mechanisms underlying PCB- and dioxin-related carcinogenesis. Future research is needed to better understand the complex role LTL may play in cancer.

## Supplemental Material

(102 KB) PDF
