# Supplementary material for: Cross-sectional Associations between Exposure to Persistent Organic Pollutants and Leukocyte Telomere Length among U.S. Adults in NHANES, 2001–2002
Source: Environ Health Perspect. 2015 Oct 9;124(5):651–8. doi: 10.1289/ehp.1510187 (PMC4858394; doi:10.1289/ehp.1510187)
Supplement: (102 KB) PDF [file ehp.1510187.s001.acco.pdf]

Environ Health Perspect
DOI: 10.1289/ehp.1510187

Note to readers with disabilities: *EHP* strives to ensure that all journal content is accessible to all readers. However, some figures and Supplemental Material published in *EHP* articles may not conform to 508 standards due to the complexity of the information being presented. If you need assistance accessing journal content, please contact ehp508@niehs.nih.gov. Our staff will work with you to assess and meet your accessibility needs within 3 working days.

Supplemental Material

Cross-Sectional Associations between Exposure to Persistent Organic Pollutants and Leukocyte Telomere Length among U.S. Adults in NHANES, 2001-2002

Susanna D. Mitro, Linda S. Birnbaum, Belinda L. Needham, and Ami R. Zota

Table of Contents

Figure S1. Exclusion criteria used to form the study population

Table S1. Percent difference (95% CI) in leukocyte telomere length (T/S ratio) by TEQ. Each model treats values below the LOD differently

Table S2. Percent difference (95% CI) in leukocyte telomere length (T/S ratio) by non-dioxin-like PCBs, non-ortho PCBs, and TEQ (modeled as wet weights (ng/g or pg/g))

Table S3. LTL and risk/odds of dioxin- or PCB-associated cancers

References

Figure S1. Exclusion criteria used to form the study population.

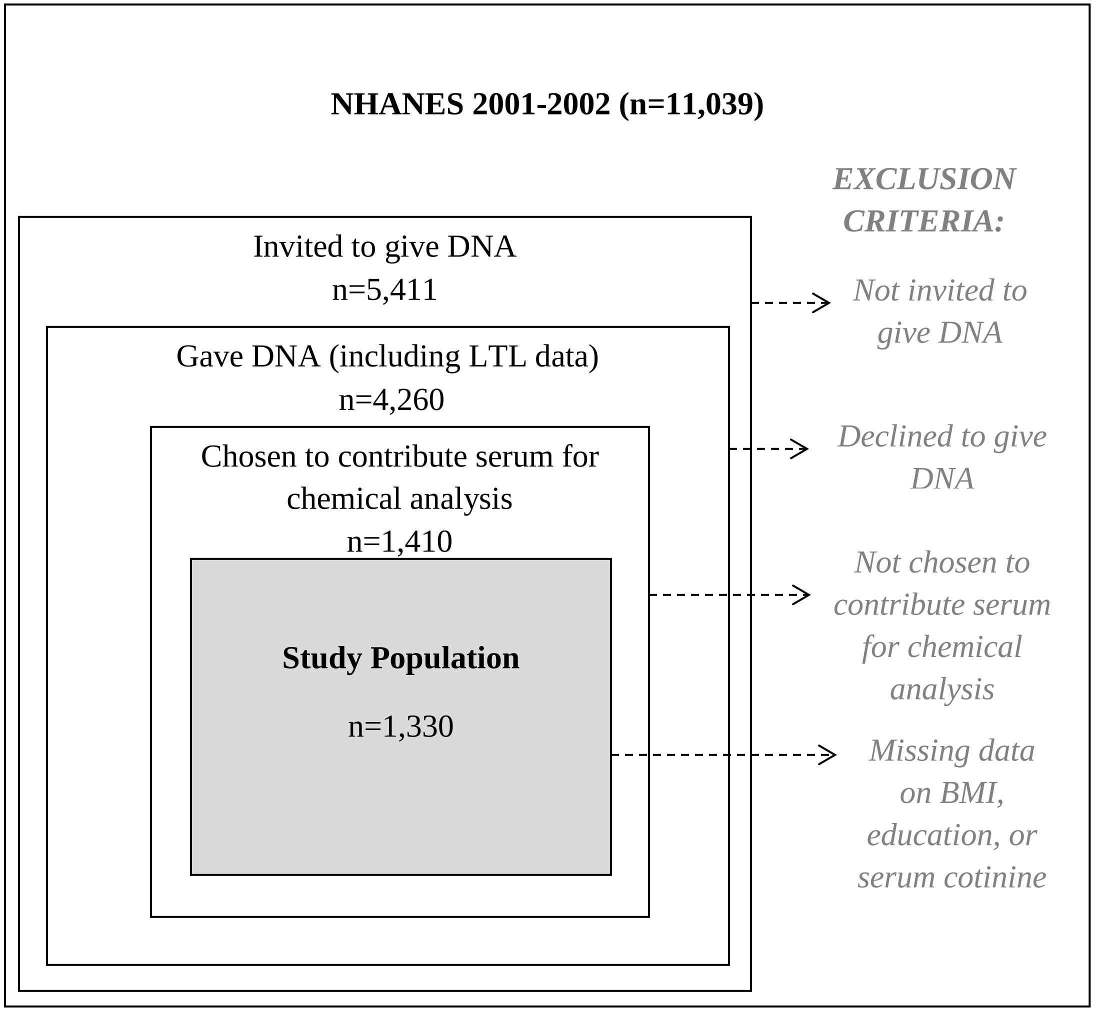

Table S1. Percent difference (95% CI) in leukocyte telomere length (T/S ratio) by TEQ. Each model treats values below the LOD differently.

**TEQ metric**	**n**	**Model 3**[a] % difference (95% CI)	**Treatment of values below the LOD**
**Final model**			
Per doubling of exposure	976	5.29 (1.66, 9.05)**	All congeners included; values <LOD modeled as LOD/√2
**Excluding <33% detected**			
Per doubling of exposure	995	4.84 (1.58, 8.20)**	Congeners with <33% of values detected are excluded
**Excluding <50% detected**			
Per doubling of exposure	996	4.93 (1.53, 8.44)**	Congeners with <50% of values detected are excluded
**Multiple imputation**			
Per doubling of exposure	976	4.30 (1.09, 7.61)*	All congeners included; values <LOD were imputed[b]

Abbreviations: TEQ: toxic equivalent; CI: confidence interval; LOD: Limit of detection

[a]Model 3 is adjusted for age, age^2, sex, race/ethnicity, BMI, (log)cotinine, white blood cells, percent lymphocytes, percent monocytes, percent neutrophils, percent eosinophils, percent basophils, serum lipids, and non-dioxin-like PCBs.

[b]We averaged 5 runs of the SAS lifereg model, assuming that the values fell between zero and $LOD/\sqrt{2}$ for each congener and that the congeners were log-normally distributed, to generate the imputed values. Only congeners with at least 10% of values detected were imputed to ensure valid imputation; for congeners with less than 10% of values detected, the sample-specific $LOD/\sqrt{2}$ was used for <LOD values.

*$P < 0.05$; ** $P < 0.01$*

Table S2. Percent difference (95% CI) in leukocyte telomere length (T/S ratio) by non-dioxin-like PCBs, non-ortho PCBs, and TEQ (modeled as wet weights (ng/g or pg/g)).

		**Model 1[a]**		**Model 2[b]**		**Model 3[c]**
**Metric**	**n**	% difference (95% CI)	**n**	% difference (95% CI)	**n**	% difference (95% CI)
**Non-dioxin-like PCBs**						
Per doubling of exposure	1265	3.89 (1.24, 6.61)**	1259	3.62 (0.93, 6.38)*	1093	0.32 (-2.34, 3.06)
Quartile 1 (≤ 0.45 ng/g)		Ref		Ref		Ref
Quartile 2 (0.46 – 0.87 ng/g)		6.37 (0.34, 12.76)*		7.04 (2.05, 12.26)**		4.36 (-0.12, 9.03)
Quartile 3 (0.88 – 1.62 ng/g)		10.87 (3.84, 18.38)**		10.88 (4.30, 17.87)**		5.49 (-0.97, 12.37)
Quartile 4 (> 1.62 ng/g)		12.35 (2.91, 22.66)*		11.84 (2.61, 21.90)*		3.02 (-5.49, 12.31)
P trend		*0.013**		*0.022**		*0.61*
**Non-ortho PCBs**						
Per doubling of exposure	1134	4.21 (2.44, 6.01)**	1128	4.22 (2.75, 5.72)**	1093	3.68 (2.08, 5.31)**
Quartile 1 (≤ 0.17 pg/g)		Ref		Ref		Ref
Quartile 2 (0.18 – 0.29 pg/g)		3.51 (-1.93, 9.26)		4.27 (-0.82, 9.61)		4.30 (-0.89, 9.75)
Quartile 3 (0.30 – 0.48 pg/g)		6.10 (-0.18, 12.77)		6.55 (0.87, 12.55)*		5.31 (0.58, 10.26)*
Quartile 4 (> 0.48 pg/g)		11.54 (4.31, 19.27)**		11.66 (5.04, 18.71)**		8.78 (2.08, 15.93)*
P trend		*0.002***		*0.001***		*0.0099***
**TEQ**						
Per doubling of exposure	1002	5.58 (2.36, 8.90)**	998	5.70 (2.40, 9.10)**	975	5.20 (1.74, 8.79)**
Quartile 1 (≤ 0.08 pg/g)		Ref		Ref		Ref
Quartile 2 (0.09 – 0.11 pg/g)		3.97 (-2.48, 10.85)		4.68 (-0.78, 10.43)		4.30 (-1.07, 9.96)
Quartile 3 (0.12 – 0.18 pg/g)		6.97 (-0.34, 14.83)		7.94 (1.48, 14.82)*		6.91 (0.37, 13.88)*
Quartile 4 (> 0.18 pg/g)		9.22 (0.04, 19.25)*		9.29 (0.51, 18.83)*		6.95 (-1.41, 16.02)
P trend		*0.044**		*0.029**		*0.073*

Abbreviations: PCB: polychlorinated biphenyl; TEQ: toxic equivalent; CI: confidence interval; Ref: reference category

[a]Model 1 is adjusted for age, age^2, serum lipids

[b]Model 2 is adjusted for age, age^2, sex, race/ethnicity, BMI, (log)cotinine, white blood cells, percent lymphocytes, percent monocytes, percent neutrophils, percent eosinophils, percent basophils, serum lipids.

[c]Model 3 is additionally adjusted for a second exposure metric. Non-dioxin-like PCBs are adjusted for non-ortho PCBs, serum lipids. Non-ortho PCBs and TEQ are adjusted for non-dioxin-like PCBs. *$P < 0.05$; ** $P < 0.01$

Table S3. LTL and risk/odds of dioxin- or PCB-associated cancers.

Reference	Cases	Cancer Type	Study design	Association between LTL and cancer risk/odds
(Fu et al. 2012)	140	Liver	Retrospective, Case-control	Positive (quartile P_{trend}=0.079)
(Liu et al. 2011)	240	Liver	Retrospective, Case-control	Positive (quartile P_{trend} < 0.001)
(Anic et al. 2013)	198	Melanoma	Retrospective, Case-control	Positive (tertile P_{trend} < 0.0001)
(Burke et al. 2013)	119	Melanoma	Retrospective, Case-control	Positive (short LTL OR: 2.81 (95% CI: 1.02–7.72), P = 0.04)
(Han et al. 2009)	218	Melanoma	Prospective, Case-control	Positive (quartile P_{trend} = 0.09)
(Nan et al. 2011)	557	Melanoma	Prospective, Case-control	Positive (quartile P_{trend} = 0.0003)
(Sun et al. 2015)	191	Lung	Retrospective, Case-control	Positive among participants > 60 years (long LTL OR: 0.52 (95% CI: 0.29–0.94), P = 0.031)
(Kim et al. 2015)	151	Lung	Prospective, Longitudinal	LTL positively associated with recurrence in women (long LTL hazard ratio: 2.25 (1.02–4.96; P = 0.044)
(Seow et al. 2014)	847	Lung	Prospective, Case-control	Positive (quartile P_{trend} < 0.0001)
(Sanchez-Espiridion et al. 2014)	1385	Lung	Retrospective, Case-control	Positive for adenocarcinoma (quartile P_{trend} < 0.001)
(Lan et al. 2013)	215	Lung	Prospective, Case-control	Positive (tertile P_{trend} = 0.003)
(Shen et al. 2011)	229	Lung	Prospective, Case-control	Positive (quartile P_{trend} = 0.05)
(Hosgood et al. 2009)	120	Lung	Retrospective, Case-control	No association (Shortest vs longest quartile OR: 1.58 (95% CI: 0.79–3.18)

(Jang et al. 2008)	243	Lung	Retrospective, Case-control	Inverse (quartile P_{trend} < 0.0001)
(Wu et al. 2003)	54	Lung	Retrospective, Case-control	Inverse (quartile P_{trend} = 0.002)
(Hosnijeh et al. 2014)	235	NHL	Prospective, Case-control	Positive for follicular lymphoma and diffuse large B-cell lymphoma (quartile P_{trends} = 0.02, 0.03 respectively)
(Lan et al. 2009)	107	NHL	Prospective, Case-control	Positive (quartile P_{trend} = 0.003)
(Widmann et al. 2007)	40	NHL	Retrospective, Case-control	Inverse (shortest vs longest quartile OR: 19.0 (95% CI: 2.1-170.4))
(Lee et al. 2003)	15	NHL	Retrospective, Case-control	Inverse (Wilcoxon test P < 0.001)
(Xie et al. 2013)	137	Soft tissue sarcoma	Retrospective, Case-control	Positive (quartile P_{trend} < 0.001)

Abbreviations: PCB: polychlorinated biphenyl; Prospective study (LTL measured before outcome had developed); Retrospective study (LTL measured after outcome had developed); NHL: Non-Hodgkin's lymphoma; LTL: Leukocyte telomere length; OR: Odds ratio

References

Anic GM, Sondak VK, Messina JL, Fenske NA, Zager JS, Cherpelis BS, et al. 2013. Telomere length and risk of melanoma, squamous cell carcinoma, and basal cell carcinoma. Cancer Epidem 37:434-439.

Burke LS, Hyland PL, Pfeiffer RM, Prescott J, Wheeler W, Mirabello L, et al. 2013. Telomere length and the risk of cutaneous malignant melanoma in melanoma-prone families with and without CDKN2A mutations. PLOS ONE 8:e71121.

Fu X, Wan S, Hann HW, Myers RE, Hann RS, Au J, et al. 2012. Relative telomere length: A novel non-invasive biomarker for the risk of non-cirrhotic hepatocellular carcinoma in patients with chronic Hepatitis B infection. Eur J Cancer 48:1014-1022.

Han J, Qureshi AA, Prescott J, Guo Q, Ye L, Hunter DJ, et al. 2009. A prospective study of telomere length and the risk of skin cancer. J Invest Dermatol 129:415-421.

Hosgood HD 3rd, Cawthon R, He X, Chanock S, Lan Q. 2009. Genetic variation in telomere maintenance genes, telomere length, and lung cancer susceptibility. Lung Cancer 66:157-161.

Hosnijeh FS, Matullo G, Russo A, Guarrera S, Modica F, Nieters A, et al. 2014. Prediagnostic telomere length and risk of B-cell lymphoma-results from the EPIC cohort study. Int J Cancer 135:2910-2917.

Jang JS, Choi YY, Lee WK, Choi JE, Cha SI, Kim YJ, et al. 2008. Telomere length and the risk of lung cancer. Cancer Sci 99:1385-1389.

Kim ES, Ye Y, Vaporciyan AA, Xing J, Huang M, Gu J, et al. 2015. Telomere length and recurrence risk after curative resection in patients with early-stage non-small-cell lung cancer: A prospective cohort study. J Thorac Oncol 10:302-308.

Lan Q, Cawthon R, Shen M, Weinstein SJ, Virtamo J, Lim U, et al. 2009. A prospective study of telomere length measured by monochrome multiplex quantitative PCR and risk of non-Hodgkin lymphoma. Clin Cancer Res 15:7429-7433.

Lan Q, Cawthon R, Gao Y, Hu W, Hosgood HD 3rd, Barone-Adesi F, et al. 2013. Longer telomere length in peripheral white blood cells is associated with risk of lung cancer and the rs2736100 (CLPTM1L-TERT) polymorphism in a prospective cohort study among women in China. PLOS ONE 8:e59230.

Lee JJ, Nam CE, Cho SH, Park KS, Chung IJ, Kim HJ. 2003. Telomere length shortening in non-Hodgkin's lymphoma patients undergoing chemotherapy. Ann Hematol 82:492-495.

Liu J, Yang Y, Zhang H, Zhao S, Liu H, Ge N, et al. 2011. Longer leukocyte telomere length predicts increased risk of Hepatitis B virus-related hepatocellular carcinoma: A case-control analysis. Cancer 117:4247-4256.

Nan H, Du M, De Vivo I, Manson JE, Liu S, McTiernan A, et al. 2011. Shorter telomeres associate with a reduced risk of melanoma development. Cancer Res 71:6758-6763.

Sanchez-Espiridion B, Chen M, Chang JY, Lu C, Chang DW, Roth JA, et al. 2014. Telomere length in peripheral blood leukocytes and lung cancer risk: A large case-control study in Caucasians. Cancer Res 74:2476-2486.

Seow WJ, Cawthon RM, Purdue MP, Hu W, Gao YT, Huang WY, et al. 2014. Telomere length in white blood cell DNA and lung cancer: A pooled analysis of three prospective cohorts. Cancer Res 74:4090-4098.

Shen M, Cawthon R, Rothman N, Weinstein SJ, Virtamo J, Hosgood HD 3rd, et al. 2011. A prospective study of telomere length measured by monochrome multiplex quantitative PCR and risk of lung cancer. Lung Cancer 73:133-137.

Sun B, Wang Y, Kota K, Shi Y, Motlak S, Makambi K, et al. 2015. Telomere length variation: A potential new telomere biomarker for lung cancer risk. Lung Cancer 88:297-303.

Widmann TA, Herrmann M, Taha N, Konig J, Pfreundschuh M. 2007. Short telomeres in aggressive non-Hodgkin's lymphoma as a risk factor in lymphomagenesis. Exp Hematol 35:939-946.

Wu X, Amos CI, Zhu Y, Zhao H, Grossman BH, Shay JW, et al. 2003. Telomere dysfunction: A potential cancer predisposition factor. J Natl Cancer I 95:1211-1218.

Xie H, Wu X, Wang S, Chang D, Pollock RE, Lev D, et al. 2013. Long telomeres in peripheral blood leukocytes are associated with an increased risk of soft tissue sarcoma. Cancer 119:1885-1891.
